# The effect of passive music listening in addition to conventional physiotherapy on pain, anxiety, and quality of life in patients with chronic neck pain

**DOI:** 10.1590/1806-9282.20250297

**Published:** 2025-12-05

**Authors:** Mustafa Savas Torlak, Emine Atici, Osman Tufekci, Osman Karaca, Burcu Dursun, Elif Tunc

**Affiliations:** 1 KTO Karatay University, Vocational School of Health Services, Department of Physical Therapy – Konya, Turkey.; 2 Istanbul Okan University, College of Health Sciences, Department of Physiotherapy and Rehabilitation – Istanbul, Turkey.; 3 Farabi Hospital, Department of Physical Therapy – Konya, Turkey.; 4 KTO Karatay University, College of Health Sciences, Department of Physiotherapy and Rehabilitation – Konya, Turkey.

**Keywords:** Anxiety, Chronic pain, Music, Physical therapy modalities

## Abstract

**OBJECTIVE:**

The aim of this study was to investigate the effect of passive music listening in addition to conventional physiotherapy on pain, anxiety, and quality of life in patients with chronic neck pain.

**METHODS:**

The study included 40 people aged 30–50 years with chronic neck pain, who volunteered to participate in it. The participants were randomized into a control group (n=20) and a music group (n=20). Participants in the control group received a classical physiotherapy programme, 5 days a week for 4 weeks. Participants in the music group listened to music through headphones throughout the treatment.

**RESULTS:**

At the end of treatment, there was a statistical difference in the Beck Anxiety Inventory, visual analog scale, Neck Disability Index, and SF-36 physical scores in intragroup comparisons in both groups (p<0.05), When comparing the differences between the groups before and after treatment, it was found that the decrease in visual analog scale score of the participants in the music group was statistically significant compared to the decrease in visual analog scale score of the participants in the control group (p<0.01).

**CONCLUSION:**

Passive listening to music has been shown to have a positive effect on chronic neck pain when used in addition to conventional physiotherapy.

## INTRODUCTION

Neck pain is a very common condition that causes pain, disability, and economic costs^1^. Neck pain can be caused by many factors including age, gender, sleep problems, anxiety, depression, work-related factors, neuromusculoskeletal diseases, autoimmune diseases, and genetics^2^. There are pharmacological and non-pharmacological treatment options in the treatment of neck pain^3^.

Passive music listening is a noninvasive, easy-to-apply, and cost-effective therapy that has been shown to relieve pain in a variety of pain disorders^4,5^. Music is thought to modulate pain by triggering the release of endogenous opioids such as β-endorphins, which stimulate descending pain modulatory pathways in the brainstem and spinal cord to suppress nociceptive stimuli^6^. It is also reported that music listening increases dopamine release from the caudate and nucleus accumbens, and dopamine has a role in central analgesia^7^.

The aim of this study was to investigate the effect of passive music listening in addition to conventional physiotherapy on pain, anxiety, and quality of life in patients with chronic neck pain.

## METHODS

### Patients

The study was conducted in a private hospital between February 2024 and July 2024. The study included 40 volunteer patients, aged 30–50 years, who were diagnosed by a physical medicine and rehabilitation physician as having chronic neck pain without neurological deficits (e.g., neck trauma, radiculopathy, neck instability). Patients were randomly divided into control (n=20) and music groups (n=20). Inclusion criteria included patients between 30 and 50 years of age, with neck pain for more than 3 months, without any neurological deficit, and with a pain intensity of 5 or more on a visual analog scale (VAS). Patients who regularly took painkillers or antidepressants and cortisone, those with serious chronic conditions, and those who had spinal surgery were excluded from the study.

### Randomization

The trial was designed as a single-blind, randomized, prospective trial. Two different treatment options for randomization were created on a computer by a statistician and numbered from largest to smallest by placing them in envelopes. Notably, 40 patients were divided into two groups using the sequential randomization method. The person administering the treatment opened the envelopes in turn and applied the treatment in the envelope according to the number of patients. In the present study, statistical blinding was the only type of blinding performed, as it was not possible to blind the patients or the practitioner.

### Ethical consideration

The study was approved by the Ethics Committee of Istanbul Okan University of Science, Social and Non-Invasive Health Sciences Research (number: 172-15). The study was registered on ClinicalTrials.gov (NCT06285383). All patients were given verbal and written information about the study and signed an informed consent form. The trial was conducted in accordance with the ethical principles of the Declaration of Helsinki. Participants gave their informed consent to be included in this study.

### Treatment

Participants in the control group received a conventional physiotherapy programme, 5 days a week for 4 weeks. This programme included ultrasound (5 min, 1.5 w/cm^2^, pulsed), hot pack (20 min), and TENS (20 min, 60–120 Hz frequency). In addition, patients were given an appropriate exercise programme after assessment by a physiotherapist. These exercises were to be done as a home programme^8^.

The participants in the music group listened to a piece of music called Pachelbel Canon D major during the conventional physiotherapy programme, with no outside noise (70 dB through headphones). This piece of music was chosen because it has been shown in other studies to reduce anxiety and stimulate the parasympathetic nervous system^9^.

### Outcomes

Before and after the study, the VAS was used to assess neck pain intensity, the Beck Anxiety Inventory (BAI) was used to assess anxiety level, the Neck Disability Index (NDI) was used to assess disability, and the Short Form-36 (SF-36) was used to assess quality of life. In addition, a 6-item form questioning demographic characteristics was completed by the participants before the study.

VAS is a practical and easy-to-use scale for subjective assessment of pain. A 100 mm straight line is used for measurement. The patient is asked to mark the intensity of the pain felt on this line. On the line, "0" means no pain and "100" means excruciating pain^10^.

The BAI is a 4-point Likert-type rating scale consisting of 21 items and scored between 0 and 3 and raw scores ranging from 0 to 63. The higher the total score, the higher the anxiety symptoms experienced by the person^11^.

The NDI consists of 10 parts: pain intensity, personal care, lifting, reading, headaches, concentration, working, driving, sleeping, and entertainment. Each item is scored from 0 to 5^12^.

The SF-36 assesses a person's general health. It consists of a total of 36 questions assessing two basic parameters (mental and physical) and eight sub-parameters. Each parameter can be scored from 0 to 100 points. High scores indicate a good quality of life^13^.

### Sample size

The sample size calculation was done using G*power analysis software Version 3.0.10 (G*Power, Franz Faul, Universitat Kiel, Germany). It was calculated according to the previous study on chronic neck pain^14^. VAS scores were used to estimate the sample size. The analysis indicated that 20 participants for each group were enough to detect a large Cohen's effect (d=0.93) with an alpha error probability of 0.05 and a power of 80%.

### Statistical analysis

Data from study participants were analyzed using the IBM SPSS Statistics 23 package. Categorical data were expressed as frequencies and percentages. Numerical data showing normal distribution were expressed as mean±standard deviation, while data not showing normal distribution were expressed as median interquartile range (IQR). Chi-square test and Fisher's exact test were used to compare categorical data between groups. Wilcoxon signed-rank tests were used for within-group comparisons. Independent samples test and Mann-Whitney U test were used for comparisons between groups. p<0.05 was considered statistically significant.

## RESULTS

### Demographic data

A total of 40 individuals, 20 females and 20 males, were included in this study. The mean age of the participants was 39.12±11.15 years. The demographic data of the participants are shown in Table 1.

**Table 1:** Demographic data of the participants.

		Music group	Control group	p-value
		Mean±SD	Mean±SD	
Age (year)		41.1±12.3	37.1±9.8	0.27^a^
		n (%)	n (%)	
Sex	Women	10 (50%)	10 (50%)	1.00^b^
	Men	10 (50%)	10 (50%)	
Marital status	Married	17 (85%)	7 (35%)	0.001^b^ **
	Single	3 (15%)	13 (65%)	
Alcohol consumption	Yes	15 (75%)	5 (25%)	0.002^b^ **
	No	5 (25%)	15 (75%)	
Smoking	Yes	8 (40%)	9 (45%)	0.749^b^
	No	12 (60%)	11 (55%)	
Chronic illness	Yes	17 (85%)	15 (75%)	0.695^c^
	No	3 (15%)	5 (25%)	

a Independent samples test.

b Chi-square test.

c Fisher's exact test. SD: standard deviation.

** p<0.01.

### Within-group comparison of pre-treatment and post-treatment values

When the results of the participants’ pre-treatment and post-treatment assessments were analyzed within groups, it was found that the VAS, NDI, and BAI data of the participants in the music group decreased significantly (p<0.05), and the physical function, pain, and general health scores of the SF-36 sub-parameters increased significantly (p<0.05) (Table 2).

**Table 2:** Within-group comparison of pre- and post-treatment values.

	Music group				Control group			
	Pre-treatment	Post-treatment	p-value	r	Pre-treatment	Post-treatment	p-value	r
	Median (IQR)	Median (IQR)			Median (IQR)	Median (IQR)		
Visual analog scale	8.0 (7.3–8.0)	3.5 (2.3–5.0)	0.001**	0.88	6.5 (5.0–8.0)	4.0 (4.0–5.0)	0.001**	0.79
Neck Disability Index	37.0 (24.5–56.0)	20.0 (14.5–32.0)	0.005**	0.62	55.0 (29.0–63.0)	29.0 (14.0–49.5)	0.001**	0.72
Beck Anxiety Inventory	7.0 (4.3–14.0)	6.0 (3.5–8.8)	0.018*	0.53	8.5 (6.0–14.5)	5.0 (3.0–12.5)	0.001**	0.84
SF-36 physical function	25.0 (5.0–53.8)	72.5 (26.3–90.0)	0.001**	0.76	37.5 (3.8–58.8)	67.5 (42.5–85.0)	0.001**	0.76
SF-36 physical role	0.0 (0.0–25.0)	30.0 (0.0–75.0)	0.194	0.29	50.0 (0.0–68.8)	100.0 (75.0–100.0)	0.001**	0.74
SF-36 emotional role	0.0 (0.0–58.4)	33.3 (0.0–100.0)	0.165	0.31	33.3 (8.3–66.7)	100.0 (66.7–100.0)	0.002**	0.69
SF-36 vitality	35.0 (25.0–45.0)	40.0 (15.0–53.8)	0.643	0.10	47.5 (36.3–57.5)	50.0 (27.5–60.0)	0.937	0.02
SF-36 mental health	52.0 (36.0–63.0)	56.0 (37.0–63.0)	0.846	0.04	56.0 (48.0–60.0)	54.0 (44.0–60.0)	0.473	0.16
SF-36 social function	50.0 (25.0–75.0)	50.0 (28.1–62.5)	0.781	0.06	50.0 (28.1–50.0)	56.3 (50.0–62.5)	0.062	0.41
SF-36 pain	40.0 (22.5–55.0)	55.0 (35.0–67.5)	0.023*	0.50	50.0 (20.0–67.5)	66.3 (46.9–75.0)	0.004**	0.64
SF-36 general health	47.5 (26.3–63.8)	60.0 (46.3–65.0)	0.002**	0.69	50.0 (35.0–55.0)	50.0 (40.0–55.0)	0.084	0.39

SF-36: short form-36; r: Wilcoxon effect size (Z/√N); IQR: interquartile range.

* p<0.05,

** p<0.01.

It was found that VAS, NDI, and BAI scores of participants in the control group decreased significantly (p<0.05), and physical function, physical role, emotional role, social function, and pain scores of SF-36 sub-parameters increased significantly (p<0.05) (Table 2).

### Comparison of differences between groups before and after treatment

When comparing the differences between the groups before and after treatment, it was found that the decrease in VAS score of the participants in the music group was statistically significant compared to the decrease in VAS score of the participants in the control group (d=1.33, p<0.01) (Table 3).

**Table 3:** Comparison of differences between groups before and after treatment.

	Music group	Control group	p^m^-value	r
	Median (IQR 25/75)	Median (IQR 25/75)		
Visual analog scale	-4.00 (-5.00/-3.00)	-2.5 (-4.00/-1.00)	0.001**	0.54
	**Mean**±**SD**	**Mean**±**SD**	**p** a **-value**	**D**
Neck Disability Index	-14.20±20.31	-16.00±13.84	0.745	0.10
	**Median (IQR 25/75)**	**Median (IQR 25/75)**	**p** m **-value**	**r**
Beck Anxiety Inventory	-1.50 (-5.00/0.75)	-2.00 (-4.75/-2.00)	0.400	0.13
SF-36 physical function	20.00 (5.00/55.00)	20.00 (1.25/45.00)	0.724	0.06
SF-36 physical role	0.00 (0.00/50.00)	37.50 (0.00/75.00)	0.083	0.27
SF-36 emotional role	0.00 (0.00/66.70)	33.40 (0.00/66.70)	0.155	0.22
SF-36 vitality	0.00 (-5.00/12.50)	2.50 (-8.75/8.75)	0.967	0.01
SF-36 mental health	0.00 (-4.00/4.00)	-2.00 (-8.00/4.00)	0.493	0.11
SF-36 social function	0.00 (0.00/12.50)	12.50 (0.00/12.50)	0.110	0.25
SF-36 pain	15.03 (0.00/22.50)	5.00 (0.00/34.38)	0.944	0.01
SF-36 general health	5.00 (0.00/15.00)	5.00 (0.00/5.00)	0.154	0.23

a Independent samples test.

m Mann-Whitney U test.

SF-36: short form-36; SD: standard deviation; IQR: interquartile range; r: Mann-Whitney U test effect size (Z/√N); d: Cohen's d (effect size).

** p<0.01.

## DISCUSSION

As no single treatment has a consistent benefit in chronic pain, multimodal therapies are recommended^15^. In this study, similar to other studies, we applied many treatment modalities such as electrotherapy, heat application, ultrasound, exercise, and listening to music in chronic pain. TENS is the noninvasive transcutaneous application of electrical stimulation to produce analgesia. It is an inexpensive and reliable application used as an adjunct treatment for musculoskeletal pain in clinical settings^16^. A recent systematic review reported that TENS was effective in reducing pain intensity as an adjunctive treatment for people with neck pain^17^. It has been stated that therapeutic ultrasound is an effective and reliable treatment option for neck pain and can be applied with other treatment options^18^. Similar to the present study, improvement was observed in VAS, NDI, BAI, and SF-36 physical scores in both groups.

Exercise therapy is recognized as the best evidence-based approach to managing chronic neck pain. However, it was noted that it is important to tailor the exercise prescription to the individual with chronic neck pain^19^. In the present study, the exercise prescription was individualized by an expert physiotherapist, similar to the literature.

The main finding of the study was that the VAS value of the participants in the music group decreased statistically significantly after the treatment compared to the VAS value of the control group. Similar to the present study, a study by Kullich et al. of 65 people with chronic low back pain, divided into music and control groups, found that music had a positive effect on pain, sleep, and quality of life^20^. In another pilot study, 20 older people with chronic low back pain were given their favorite music through headphones twice a day for 4 days and found that it reduced their pain^21^. Music-induced analgesia has been shown to be effective in experimental, acute, and chronic pain in several studies^22^. Until recently, however, how music works to relieve pain has remained elusive. Studies in healthy people have shown that listening to music stimulates the prefrontal cortex, amygdala, cingulate cortex, and insula, which have strong associations with pain^23^. In addition to directly modulating nociceptive mechanisms, music can relieve pain by improving anxiety and depression. Neuroimaging studies have shown that pleasurable music increases dopamine release by stimulating the mesolimbic dopaminergic system^23^. The link between dopamine deficiency and chronic pain is well known, and dopamine has been shown to play a role in modulating anxiety^24^. In a randomized controlled study, it was found that anxiety and pain levels decreased in postoperative patients who listened to their favorite music^25^. Similarly, in the present study, it was found that pain and anxiety levels decreased in the music group.

Our study had some limitations. First, only one piece of music was played to the patients. In some trials with music, they played different music or the patients’ own music preferences. In our study, the reason for the difference between the groups in pain could only be the choice of music. Second, our results are limited by a small sample size, which limits the generalizability of our findings, but provides important findings on feasibility and acceptability to inform future studies. Furthermore, baseline differences in marital status and alcohol consumption may have influenced the outcomes. However, ANCOVA could not be applied due to the non-normal distribution of the data.

## CONCLUSION

In this study, we have shown that listening to music in addition to conventional physiotherapy reduces pain and is feasible and acceptable in individuals with chronic neck pain.

## Data Availability

The datasets generated and/or analyzed during the current study are available from the corresponding author upon reasonable request.
